# Supplementary material for: Feeding and Management of Horses with and without Free Faecal Liquid: A Case–Control Study
Source: Animals (Basel). 2021 Aug 30;11(9):2552. doi: 10.3390/ani11092552 (PMC8465618; doi:10.3390/ani11092552)
Supplement: Supplementary file 1 [file animals-11-02552-s001.zip › Supplementary files/Supplementary files/Table S2.pdf]

Table S2. Basic information on horses with (case, n = 50) and without (control, n = 50) free faecal liquid and p-values from Chi^2-test. Case and control horses within each pair were located on the same farm

Characteristics		Case, n (%)	Control, n (%)	*p-value*
Sex				0.32
	Gelding	27 (54)	22 (44)	
	Mare	23 (46)	28 (56)	
Breed type				0.14
	Warmblood horses (and crossbreds)	21 (42)	19 (38)	
	Cold-blood horses (and crossbreds)	14 (28)	8 (16)	
	Hot-blooded horses	4 (8)	3 (6)	
	Native breeds	11 (22)	20 (40)	
Coat colour				0.90
	Grey	3 (6)	7 (14)	
	Bay	22 (44)	13 (26)	
	Black	5 (10)	12 (24)	
	Chestnut	11 (22)	9 (18)	
	Paint/piebald	2 (4)	2 (4)	
	Other (Palomino/Isabelline, Leopard pattern, Buckskin, Cremello)	7 (14)	7 (14)	
Training discipline performed[1]				0.07
	Dressage	22 (44)	17 (34)	
	Show jumping	13 (26)	11 (22)	
	Leisure riding	32 (64)	34 (68)	
	Riding school	5 (10)	7 (14)	
	Breeding show	3 (6)	3 (6)	
	Breeding	5 (10)	6 (12)	
	Racing	4 (8)	10 (20)	
	Companion	10 (20)	1 (2)	
	Academic art of riding	5 (10)	8 (16)	
	Breaking in	4 (8)	5 (10)	
	Other (Western, Working equitation, Mounted archery, cross country)	7 (14)	8 (16)	
Training intensity				0.60

Very low (maximum 30 min/day, 1-3 times/week, mainly walking)	12 (24)	11 (22)	
Low (leisure riding, 30–60 min per day, 4–7 days/week, all gaits)	22 (48)	23 (44)	
Medium (riding school, more intense leisure riding, all gaits)	10 (20)	9 (18)	
High (cross country, high-level show jumping, all gaits)	1 (2)	1 (2)	
Very high (trotting and racing, high-level cross country training, endurance)	1 (2)	1(2)	
Breaking in	4 (8)	5 (10)	
Body condition score, BCS[2]			0.68
<3	11 (22)	5 (10)	
3	26 (52)	33 (66)	
>3	13 (26)	12 (24)	
Ability of horse to keep desired BCS			0.35
Easy keeper	17 (34)	17 (34)	
Normal	24 (48)	30 (60)	
Hard keeper	9 (18)	3 (6)	

[1]Multiple disciplines could be selected in the questionnaire, resulting in the sum of horses for each discipline potentially exceeding the total number of horses in the study. [2] Body condition score according to Carroll and Huntington (1988).
